# Mitochondrial Deoxyguanosine Kinase Regulates NAD^+^ Biogenesis Independent of Mitochondria Complex I Activity

**DOI:** 10.3389/fonc.2020.570656

**Published:** 2020-12-18

**Authors:** Lei Sang, Ying-Jie He, Jiaxin Kang, Hongyi Ye, Weiyu Bai, Xiao-Dong Luo, Jianwei Sun

**Affiliations:** ^1^State Key Laboratory for Conservation and Utilization of Bio-Resources in Yunnan, Center for Life Sciences, School of Life Sciences, Yunnan University, Kunming, China; ^2^Key Laboratory of Medicinal Chemistry for Natural Resource, Ministry of Education and Yunnan Province, School of Chemical Science and Technology, Yunnan University, Kunming, China

**Keywords:** deoxyguanosine kinase, NAD^+^, NMNAT2, mitochondria complex I, lung adenocarcinoma

## Abstract

Overexpression of DGUOK promotes mitochondria oxidative phosphorylation and lung adenocarcinoma progression. However, the role and mechanism of DGUOK in regulation of mitochondria function and lung cancer progression still poorly understood. Here we demonstrated that *DGUOK* regulated NAD^+^ biogenesis. Depletion of the *DGUOK* significantly decreased NAD^+^ level. Furthermore, knockout of the *DGUOK* considerably reduced expression of the *NMNAT2*, a key molecule controlling NAD^+^ synthesis, at both mRNA and protein levels. Ectopic expression of the *NMNAT2* abrogated the effect of knockdown of *DGUOK* on NAD^+^. Notably, this regulation is independent of DGUOK -mediated mitochondria complex I activity. We also showed that NMNAT2 was highly expressed in lung adenocarcinoma and negatively correlated with the patient overall survival. Our study suggested that DGUOK regulates NAD^+^ in a NMNAT2 dependent manner and DGUOK-NMNAT2-NAD^+^ axis could be a potential therapeutic target in lung adenocarcinoma.

## Introduction

Mitochondrial deoxyguanosine kinase (*DGUOK*) is a rate-limiting enzyme for the salvage pathway of purine deoxynucleotide biogenesis in mitochondria (1). It has been shown that DGUOK phosphorylates purine deoxyribonucleosides in the mitochondrial matrix. In addition, this protein phosphorylates several purine deoxyribonucleoside analogs which were used in the treatment of lymphoproliferative disorders, and this phosphorylation is critical for the effectiveness of the analogs (2). Mutations in the *DGUOK* led to mitochondrial DNA (mtDNA) depletion typically in the liver and brain, causing a hepatocerebral phenotype (3). We have previously shown that DGUOK was frequently overexpressed in lung adenocarcinoma and aberrant expression of DGUOK correlated with tumor progression and patient overall survival (4). However, the role and mechanism of DGUOK in lung cancer still poorly understood.

Nicotinamide adenine dinucleotide (NAD) is a critical sirtins (SIRT) coenzyme, NAD levels are very important for regulation of SIRT activity and thus are associated with a number of cellular and biological processes including cell survival, senescence, proliferation and Parkinson's disease (5–7). It exists in both oxidized (NAD^+^) and reduced (NADH) forms. Previous studies have demonstrated that NAD^+^ was biosynthesized through two major pathways: the *de novo* and salvage pathways (8). The salvage pathway is important for the maintenance of NAD^+^ level in cancer cells and involves 2 major enzymes, one of which is phosphoribosyltransferase (NAMPT) and the other is nicotinate phosphoribosyltransferase (NAPRT) (9). NAMPT acts as a rate‐limiting enzyme in the salvage pathway and works by transferring a phosphoribosyl group to nicotinamide (NAM) to form nicotinamide mononucleotide (NMN). NMN is then converted into NAD^+^ by nicotinamide mononucleotide adenylyltransferase (NMNAT). There are three NMNAT isoforms (NMNAT1–3) with different tissues and subcellular distributions in mammals. NMNAT family members catalyze the synthesis of NAD^+^ both in the *de novo* pathway and the salvage pathway (10, 11). Recent studies have shown that NMNAT2 is involved in colorectal cancer progression (12, 13). NAPRT is involved in the synthesis of NAD^+^ from nicotinic acid (9). Cancer cells were reported to have a high rate of NAD^+^ turnover and a low ratio of cytosolic NAD^+^/NADH due to their elevated metabolic needs. However, the role and mechanism of the NAD^+^ and NMNAT2 in cancer progression is poorly understood.

In this study, we demonstrated that DGUOK regulates NAD^+^ biogenesis through NMNAT2, and this regulation is independent of DGUOK-mediated mitochondria complex I activity (4). NMNAT2 expression was increased in lung adenocarcinoma and NMNAT2 level was negatively correlated with the overall survival of patients with lung adenocarcinoma. Our results indicate that DGUOK plays a pivotal role in mitochondrial function and tumor progression through regulation of the *NMNAT*2.

## Material and Methods

### Cell Culture

Lung cancer cell line H1650, H1299, and immortalized human kidney epithelial cell HEK293T were obtained from the Moffitt Cancer Center Lung Cancer Center of Excellence cell line repository. These cell lines were free of microbial (including mycoplasma) contamination. H1650 and H1299 cells were cultured in RPMI1640 medium supplemented with 10% fetal bovine serum (FBS) and 1% penicillin/streptomycin. HEK293T cells were maintained in DMEM medium supplemented with 10% FBS and 1% penicillin/streptomycin. All the cells were cultured at 37°C in a humidified 5% CO2 incubator.

### Inhibitor Treatment

For Rotenone treatment, H1650 cells were incubated with medium containing 500 nM (Sigma, P5499) at 37°C for 10 h in a 5% CO2 incubator prior to RNA extraction.

### Plasmids

The *DGUOK* knockout was performed using pLenti CRISPR V2 vector (Addgene_52961) encoding sgRNA targeting human *DGUOK*, and the sequence targeting *DGUOK* is: 5’-CCCCGAAGGCTCTCCATCGA-3’

*NMNAT2* cDNA was subcloned into pLenti-CMV-blasticidin vector (Addgene_17486) between BamH I and XhoI sites. pLKOs encoding shRNAs for the *NDFUB8* and the *NMNAT2* were purchased from Sigma (*ndufb8*, TRCN0000318424 and *NMNAT2*, TRCN0000318425). Retroviral and lentiviral particles were packaged in HEK293T cells using the PEI transfection method and concentrated as previously described (14).

### Antibody

The following antibodies were used in this study: anti-NMNAT2 (sc-515206), anti-GAPDH (sc-32233), and anti-DGUOK (sc-398093) antibodies were from Santa Cruz. Anti-NDUFB8 (459210) was from Thermo Fisher. Anti-Mouse, HRP (7076S) were from Cell Signaling.

### Immunoblot

Cells were lyzed in SDS-NP40 buffer (50 mM Tris, pH 8.0, 150 mM NaCl, 1% NP40, 1% SDS, 1 mM protease inhibitors cocktail) on ice for 1 min. Cells were scraped from the plate and sonicated briefly 3 times. Then lysates were heated at 95°C for 5 min and centrifuged at 20,000 × g at 4°C for 10 min. 50 μg total cell lysates were separated on SDS-PAGE and then transferred onto PVDF membrane. The membranes were blocked with non-fat dry milk for 30 min at room temperature. Following washing 3 times, the blots were incubated with primary antibodies and then peroxidase-linked anti-mouse IgG (cell signaling,7076S). The bands were detected by an ECL-plus Western blotting detection system (Tanon-5200Multi).

### qRT-PCR

Cells were washed with ice-cold PBS and total RNA was extracted from using TIANGEN RNaesy Mini kit (ER501-01). Reverse transcription was performed using the Transgen cDNA synthesis kit (AT311-03). Quantitative real-time PCR (qRT-PCR) was carried out with the Bio-rad real-time PCR system using Transgene SYBR Green PCR master mix (AQ131-02).

Primers were used as follows:

*NMNAT2* Q-PCR NS: 5’-GAGGCAGATATGGAGGTGATTG-3’*NMNAT2* Q-PCR CAS: 5’-TTTTGTATTTGCGGAGTATTGAGG-3’;*NMNAT*1 Q-PCR NS: 5’-TGGGTGGAAGTTGATACATGG-3’*NMNAT*1 Q-PCR CAS: 5’-TCCAGGCCTTTCTAGAGTAGG -3’*NRK1* Q-PCR NS: 5’- GACTCTCCGGGATACTTTGATG-3’*NRK1* Q-PCR CAS: 5’- CCTCTTCAGATTTTGTTCCATCC -3’*NAMPT* Q-PCR NS: 5’- GCTGCCACCTTATCTTAGAGTT -3’*NAMPT* Q-PCR CAS: 5’- CTTGTCAACTTCTGTAGCAAACC -3’*NARPT* Q-PCR NS: 5’- GTCCTCATCGTAGTCAGCAAC -3’*NARPT* Q-PCR CAS: 5’- CACCAGCTTATAGACGCCAC -3’*NADSYN1* Q-PCR NS: 5’- CAAGATACAGGCTTGGACCAG-3’*NADSYN1* Q-PCR CAS: 5’- CCGCTAGGACTTGAAACGAG -3’*GAPDH* Q-PCR NS: 5’-TGAAGGTCGGAGTCAACGG-3’,*GAPDH* Q-PCR CAS:5’-AGAGTTAAAAGCAGCCCTGGTG-3’.

### Blue Native Electrophoresis and Immunoblot

Blue native (BN) electrophoresis was performed as previously described (15). The Western blot analysis was carried out as described above.

### Cell Migration and Invasion Assay

Cell migration and invasion assays were performed as previously described (16). Migration and invasion assayed were carried out for 5 h and 12 h, respectively.

### Measurement of NAD^+^ Levels: LC/MS Method

Preparation of Cellular Extracts: DGUOK-knockout and control H1650 cells (1x10^7^/well in 10 ml) were seeded in 10 cm plates and cultured overnight. For metabolite analysis, cultural medium was discarded. After washing with PBS twice, the cells were extracted using 1 ml of cold 80% methanol. Following incubation at room temperature for 15 min, the methanol extracts were sonicated (60 W, work 3 s, interval 3 s, for 3 times) at 4°C. After centrifugation (12,000 g) at 4°C for 15 min, the supernatants were dried using the EYELA Centrifugal Concentrator (CVE-3110) at 7,000 rpm, 37°C. The samples were then dissolved with deionized water.

An Agilent 6100 LC/MSD system (Agilent Technologies, Palo Alto, CA, USA) was employed for the quantitative analysis of NAD^+^. A HILIC column (2.1 × 100 mm, 1.8 µm, Waters Corp., Tokyo, Japan) was used for the chromatographic separation of target. In short, 0.1% formic acid water (phase A) and acetonitrile (phase B) were of the solvent system with a suitable gradient elution procedure at a flow rate of 0.15 ml/min (0–10 min, 98%–2% B; 10–15 min, 2% B). The injection volume was 2.0 µl. The gas temperature was 325°C at a rate of 8 L/min, the nebulizer pressure was 35 psi, the fragmentor was 45 V. the NAD^+^ mass acquisition was performed using selective ion mode (SIM) at m/z 664.1 in positive condition. The quantitative NAD^+^ was obtained by analyzing the mass response abundance of NAD^+^ in standard solution and sample solutions. Metabolite peak areas were manually checked for consistency in retention times, compared with known standards and normalized based on protein concentrations and the resultant peak areas were subjected to metabolomic analyses by utilizing MetaboAnalyst 2.0. Relative significance in metabolite levels were analyzed using Student’s *t* test.

### Data Analysis

All the experiments were repeated three times and each experiment was performed in three replicates per sample. Data were analyzed using GraphPad Prism 6.0 (Graph-Pad Software Inc., San Diego, CA, USA), and all results were expressed as means ± SEM. Statistically significant differences were determined using Student’s t-test for two-group analysis. Statistical significance was defined as *P < 0.05, **P < 0.01 or ***P < 0.001.

## Result

### DGUOK Regulates NAD^+^ Biogenesis

**Figure 1:**
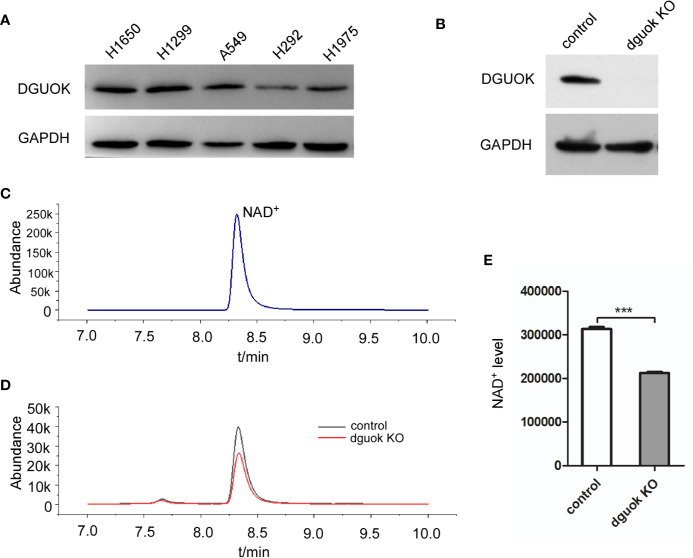
DGUOK regulates NAD^+^ biogenesis. **(A)** Western blot analysis of DGUOK level in different lung cancer cell lines. **(B)** Western blot analysis of DGUOK expression in *DGUOK*-KO and control H1650 cells. **(C)** NAD^+^ standard curve revealed by LC-MS. **(D)** LC-MS analysis of NAD^+^ level in *DGUOK*-KO and control H1650 cells. **(E)** Quantification of NAD^+^ level in *DGUOK*-KO and control H1650 cells. ***p < 0001.

We have recently reported that DGUOK overexpression promotes lung adenocarcinoma progression (4). Loss of DGUOK significantly induced mtDNA deletion and inhibited mitochondria complex I activity. Ran Jing *et al*. reported that NAD^+^ is a potential therapeutic target for mtDNA depletion syndrome, suggesting that DGUOK could involve in NAD biosynthesis (17). To test our hypothesis, we first assessed DGUOK level in a panel of lung cancer cell lines. After normalization, the results showed that DGUOK levels were higher in H1650, H1299, and A549 cells (**Figure 1A**).

To examine if DGUOK regulates NAD^+^ level, we first employed LC-MS to examine the content of NAD^+^ in *DGUOK*-KO and control H1650 cells. **Figure 1B** showed that the DGUOK protein expression was completely abrogated after infection of cells with *DGUOK*-KO lentivirus. To identify the level of NAD^+^, we first examined NAD^+^ LC-MS curve (**Figure 1B**). LC-MS analysis revealed a significant decrease in the NAD^+^ level in H1650 *DGUOK*-KO cells (**Figures 1C–E**). These results indicate a critical role of DGUOK in regulation of NAD^+^ biogenesis.

### DGUOK Regulates NMNAT2 Expression

**Figure 2:**
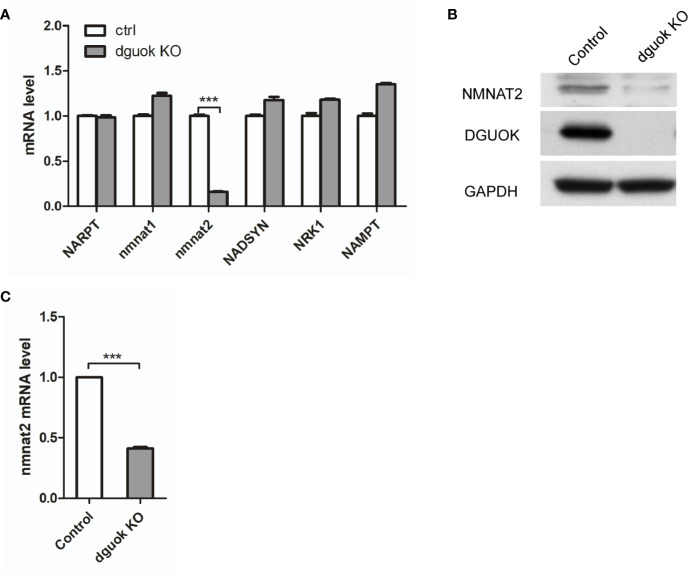
DGUOK controls NMNAT2 expression at mRNA and protein levels. **(A)** Quantitative RT-PCR analysis of the *NARPT, NMNAT1/2, NADSYN, NRK1, NAMPT* mRNA levels in *DGUOK*-KO and control H1650 cells. **(B)** Western blot analysis of DGUOK and NMNAT2 protein levels in *DGUOK*-KO and control H1650 cells. **(C)** Quantitative RT-PCR analysis of the *NMNAT2* level in *DGUOK*-KO and control H1299 cells. ***p < 0001.

NAD^+^ is synthesized through two known pathways, i.e., eight-step *de novo* cascade and the salvage pathway, in which several enzymes are involved including NAMPT, NAPRT, NRK1/2, and NMNAT1-3 (8). To investigate how DGUOK regulates NAD^+^ biogenesis, we examined the mRNA levels of these enzymes by quantitative real‐time PCR. There was no significant difference in the expression of NAMPT, NAPRT, NRK1/2, and NMNAT1-3 between *DGUOK*-KO and control H1650 cells (**Figure 2A**). Notably, we found that the mRNA levels of the *NMNAT*2 were dramatically decreased in *DGUOK*-KO cells (**Figure 2A**). Accordingly, we observed the low expression of NMNAT2 protein level in *DGUOK*-KO cells (**Figure 2B**). To further verify our results, we analyzed the mRNA level of the *NMNAT2* in *DGUOK*-KO and control H1299 cells. The results showed that depletion of DGUOK significantly inhibited the expression of the *NMNAT2* (**Figure 2C**). As NMNAT2 catalyzes the synthesis of NAD^+^ both in the *de novo* and salvage pathways. Our data indicated that NMNAT2 is likely to be a key player that bridges DGUOK and NAD^+^ biogenesis.

### DGUOK Regulates NAD^+^ Biogenesis Through NMNAT2

**Figure 3:**
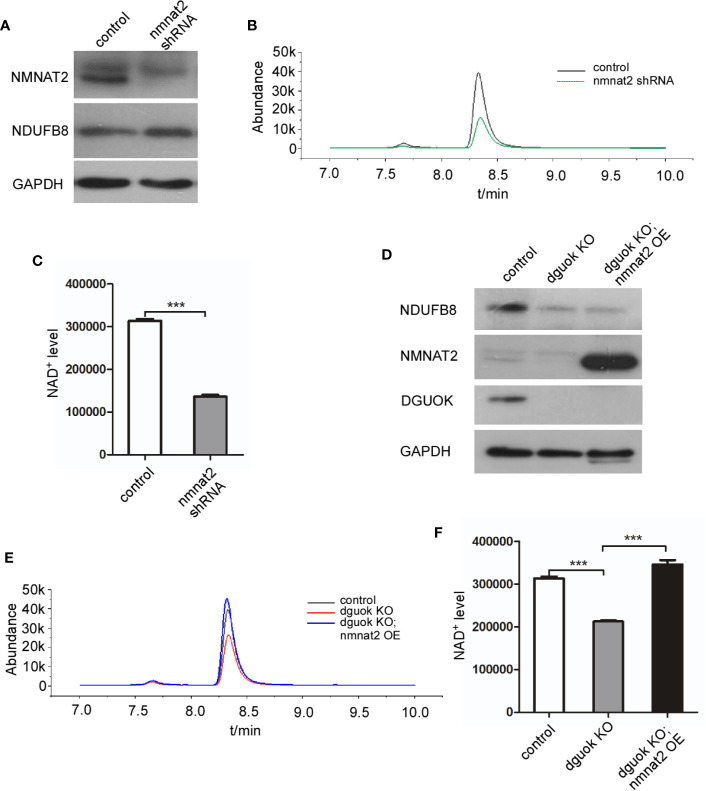
DGUOK regulates NAD^+^ biosynthesis through NMNAT*2*. **(A)** Western blot analysis of NMNAT2 and NDUFB8 expression level in *NMNAT2*-KD and control H1650 cells. **(B)** LC-MS analysis of NAD^+^ level in *NMNAT2*-KD and control H1650 cells. **(C)** Quantification of NAD^+^ level in *NMNAT2*-KD and control H1650 cells. **(D)** Western blot analysis of NMNAT2, NDUFB8 and DGUOK expression levels in *DGUOK*-KO, *DGUOK*-KO/*NMNAT2*-OE and control H1650 cells. **(E)** LC-MS analysis of NAD^+^ level in *DGUOK*-KO, *DGUOK*-KO/*NMNAT2*-OE and control H1650 cells. **(F)** Quantification of NAD^+^ level in *DGUOK*-KO, *DGUOK*-KO/*NMNAT2*-OE and control H1650 cells. ***p < 0001.

To further confirm our hypothesis, we first evaluated the effect of the *NMNAT*2 knockdown (KD) on NAD^+^ level. As expected, NAD^+^ content was reduced by 60% in *NMNAT2*-KD H1650 cells compared to their controls (**Figures 3A–C**). To investigate if DGUOK regulates NAD^+^ biogenesis through NMNAT2, we examined whether ectopic expression of NMNAT2 could rescue NAD^+^ content in *DGUOK*-KO H1650 cells. Western blot analysis revealed that NMNAT2 protein was efficiently expressed in *NMNAT2*-transfected cells (**Figure 3D**). NAD^+^ levels in *DGUOK*-KO cells were restored after ectopic expression of NMNAT2 (**Figures 3D–F**). Taken together, these data suggested that NMNAT2 mediates DGUOK regulated NAD^+^ biogenesis.

### NMNAT2 Expression Regulated by DGUOK Is Independent of Mitochondria Respiratory Complex I Activity

**Figure 4:**
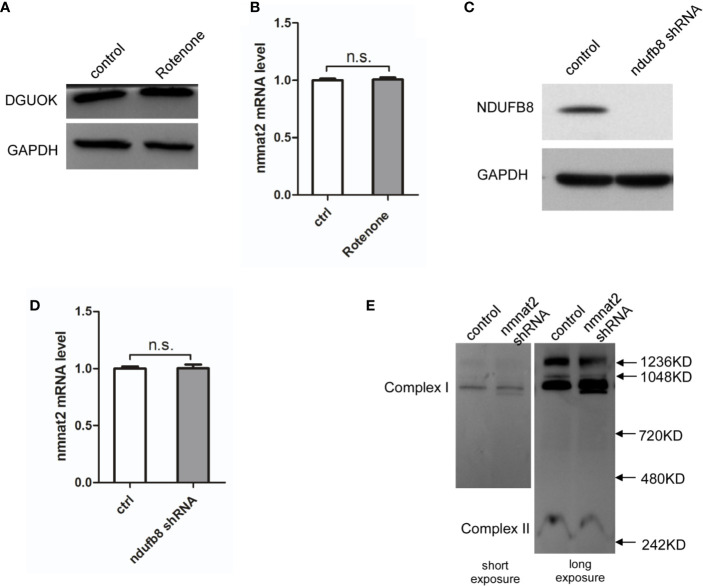
DGUOK regulates NMNAT2 independent of mitochondria complex I activity. **(A)** Western blot analysis of DGUOK in control and Rotenone treated H1650 cells. **(B)** qRT-PCR analysis of the *NMNAT2* mRNA level in Rotenone treated and control H1650 cells. **(C)** Western blot analysis of NDUFB8 in *NDUFB8*-KD and control H1650 cells. **(D)** qRT-PCR analysis of the *NMNAT2* mRNA level in *NDUFB8*-KD and control H1650 cells. **(E)** BN-PAGE gel analysis of mitochondria complex I level in *NMNAT2*-KD and control H1650 cells. n.s. not significant.

It has been shown that DGUOK is required for mtDNA maintain and mitochondria respiratory complex I activity (4). To determine whether the regulation of NAD^+^ biogenesis and NMNAT*2* expression by DGUOK is due to the mitochondrial complex I activity, we investigated the effects of Rotenone, the complex I inhibitor, on NMNAT2 and DGUOK level. As shown in **Figures 4A, B**, Rotenone treatment did not significantly reduce the expression of DGUOK protein and NMNAT2 mRNA, respectively (**Figures 4A, B**), suggesting that regulation of NMNAT2 by DGUOK is independent of mitochondria Complex I activity. To further confirm the result, we knocked down the *NDUFB8*, a nuclear genome encoded complex I subunit, to inhibit complex I level and activity (**Figure 4C**). Immunoblotting and qRT-PCR analyses showed that depletion of the *NDUFB8* severely damaged the assembly of the complex I. However, inhibition of mitochondria complex I activity by *NDUFB8* knockdown had no effect on the *NMNAT2* mRNA level (**Figure 4D**). At the same time, while NAD^+^ is necessary for the mitochondrial complex I function, we did not observe that reduction of NAD^+^ level by *NMNAT2* knockdown affected the level of mitochondrial complex I (**Figure 4E**). These results indicated that the regulation of NMNAT2 by DGUOK is independent of mitochondria respiratory complex I activity and that DGUOK controls mitochondrial function through regulation of two parallel pathways.

### NMNAT2 Is Upregulated and Correlates With Overall Survival in Patients With Lung Adenocarcinoma

**Figure 5:**
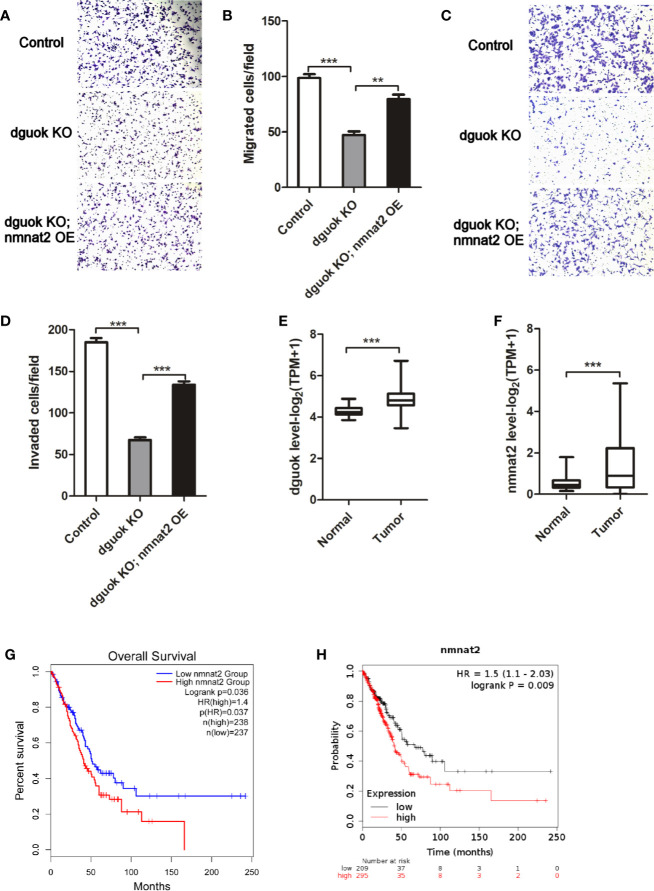
*NMNAT2* was up-regulated and correlates with poor overall survival in patients with lung adenocarcinoma. **(A)** Representative images of *in vitro* migration in control, *DGUOK*-KO and *DGUOK*-KO/NMNAT2 H1650 cells. **(B)** Ectopic expression of NMNAT2 largely rescued cell migration in *DGUOK*-KO H1650 cells. **(C)** Representative images of *in vitro* invasion in control, *DGUOK*-KO and *DGUOK*-KO/NMNAT2 H1650 cells. **(D)** Ectopic expression of NMNAT2 largely rescued cell invasion in *DGUOK*-KO H1650 cells. **(E)** DGUOK level was up-regulated in lung adenocarcinoma when compared to adjacent normal tissue. **(F)** NMNAT2 level was up-regulated in lung adenocarcinoma when compared to adjacent normal tissue. **(G, H)**. NMNAT2 expression negatively correlates with overall survival of patients with in lung adenocarcinoma in two databases: GEPIA (**G**, http://gepia.cancer-pku.cn/detail.php?gene=&clicktag=survival) and KM-Express (**H**, https://kmplot.com/analysis/). **p < 001, ***p < 0001.

We have previously shown frequent overexpression of DGUOK in lung adenocarcinoma and close association of elevated expression of DGUOK with tumor progression and patient survival (4). Knockout of the *DGUOK* in H1650 cells significantly inhibited cell migration and invasion (**Figures 5A–D**). Because DGUOK regulates *NMNAT2*, we further investigated if DGUOK regulates cell migration and invasion through NMNAT2 and NAD^+^. NMNAT2 was ectopically expressed in H1650 *DGUOK-*KO cells. We found that the expression of *NMNAT2* largely rescued the effects of depletion of DGUOK on cell migration and invasion (**Figures 5A–D**), suggesting that NMNAT2 and NAD^+^ play an important role in DGUOK-mediated cell migration and invasion. Subsequently, we evaluated mRNA level of the *DGUOK* and the *NMNAT2* in lung adenocarcinoma compared to paired adjacent normal tissues (http://gepia.cancer-pku.cn/detail.php?gene=&clicktag=survival).

We found that the *DGUOK* and the *NMNAT2* were dramatically up-regulated in lung adenocarcinoma (**Figures 5E, F**). Similar to the *DGUOK*, high levels of *NMNAT2* were associated with the poor overall survival of patients with lung adenocarcinoma (**Figure 5G**). To confirm this finding, we further analyzed the data from https://kmplot.com/analysis/and noticed that the *NMNAT2* expression was also negatively correlated with the patients’ overall survival (**Figure 5H**). Collectively, these results indicated that DGUOK-NMNAT2 axis is frequently elevated in lung adenocarcinoma and could be served as poor prognostic biomarker in this malignancy. Since NMNAT2 involved lung adenocarcinoma progression, targeting NMNAT2 may benefit in the treatments of lung adenocarcinoma. As DGUOK-NMNAT2 induces NAD^+^ biogenesis, the DGUOK-NMNAT2-NAD^+^ pathway could be potential therapeutic target in lung adenocarcinoma.

## Discussion

We have recently reported frequent overexpression of DGUOK lung adenocarcinoma (4). The upregulation of DGUOK was associated with poor overall survival of the patients with lung carcinoma. A well-known function of DGUOK is to regulate mtDNA maintenance and mitochondria complex I activity. It has been shown that DGUOK promotes the lung adenocarcinoma cancer progression and cancer stemness through mitochondria complex I activity (4). In this report, we demonstrated that DGUOK induced NMNAT2 expression at mRNA and protein levels which led to activation of NAD^+^ biogenesis. More significantly, DGUOK regulation of NMNAT2 is independent of mitochondria complex I activity. Thus, our data revealed a novel function of DGUOK, i.e., link DGUOK to NMNAT2/NAD^+^ cascade.

NAD^+^ level has been shown to be up-regulated in human cancer (18). NAD^+^ is an important coenzyme for SIRT, which involves in different cancers (19–21). Recently, accumulated evidence indicates that the NAD^+^ biosynthesis pathway plays an important role in tumor progression and metastasis (22). Ye *et al* reported that targeting the NAD^+^ salvage pathway suppressed APC mutation-driven colorectal cancer growth (23). Nicotinamide phosphoribosyl transferase (NAMPT), which is a rate-limiting enzyme for NAD^+^ synthesis in the salvage pathway, was shown to be overexpressed in many types of cancer, suggesting that NAMPT acts as a regulator of cancer invasion and metastasis (7, 24). NMNAT2, also a rate-limiting enzyme for NAD^+^ synthesis, catalyzes the synthesis of NAD^+^ both in the *de novo* pathway and the salvage pathway. NMNAT2 was shown to be a promising diagnostic and therapeutic target for colorectal cancer (12, 13) However the role of NMNAT2 in lung adenocarcinoma is unclear. In this study, we found that NMNAT2 mediates NAD^+^ biogenesis induced by DGUOK and that the NMNAT2 expression negatively correlates with overall survival in the patients with lung adenocarcinoma.

In summary, our results revealed a new pathway, i.e. DGUOK-NMNAT2-NAD^+^. DGUOK induces NAD^+^ level through NMNAT2. The regulation of NMNAT2 and NAD^+^ by DGUOK is independent of mitochondria respiratory complex I activity. Furthermore, we have shown frequent overexpression of DGUOK-NMNAT2 in lung adenocarcinoma and the association of this pathway with poor prognosis of this malignancy. Furthermore, we recently reported DGUOK promote cancer cell stemness in lung adenocarcinoma. Depletion of DGUOK significantly reduced the sphere formation in H1650 and A549 cells (4). In current study, we showed that knockout of DGUOK reduced cell migration and invasion and that ectopic expression of NMNAT2 overrode these phenotypes resulted from DGUOK knockdown (**Figure 5**). These findings suggest pro-tumorigenic potential of DGUOK. Collectively, our data indicate that the DGUOK-NMNAT2-NAD^+^ axis could be a prognostic marker and a critical therapeutic target in lung adenocarcinoma.

## Data Availability Statement

The original contributions presented in the study are included in the article/supplementary material. Further inquiries can be directed to the corresponding authors.

## Author Contributions

Study concept and design: JK, YH, and JS. Acquisition of data: LS, JK, and YH. Drafting and editing of the manuscript: LS, JK, YH, and JS. Analysis and interpretation of data: WB, HY, and JS. Critical revision of the manuscript: XL. All authors contributed to the article and approved the submitted version.

## Funding

This work was supported by National Natural Science Foundation of China (No.31671448), Yunnan Applicative and Basic Research Program (No.2019FY003030) and a grant (No.2020DAMOP-005) from NHC key Laboratory of Drug Addiction medicine.

## Conflict of Interest

The authors declare that the research was conducted in the absence of any commercial or financial relationships that could be construed as a potential conflict of interest.

## References

[1] Taanman JW Muddle JR Muntau AC Mitochondrial DNA depletion can be prevented by dGMP and dAMP supplementation in a resting culture of deoxyguanosine kinase-deficient fibroblasts. Hum Mol Genet (2003) 12(15):1839–45. 10.1093/hmg/ddg192 12874104

[2] Rodriguez CO Jr Mitchell BS Ayres M Eriksson S Gandhi V Arabinosylguanine is phosphorylated by both cytoplasmic deoxycytidine kinase and mitochondrial deoxyguanosine kinase. Cancer Res (2002) 62(11):3100–5. 12036920

[3] Mandel H Szargel R Labay V Elpeleg O Saada A Shalata A The deoxyguanosine kinase gene is mutated in individuals with depleted hepatocerebral mitochondrial DNA. Nat Genet (2001) 29(3):337–41. 10.1038/ng746 11687800

[4] Lin S Huang C Sun J Bollt O Wang X Martine E The mitochondrial deoxyguanosine kinase is required for cancer cell stemness in lung adenocarcinoma. EMBO Mol Med (2019) 11(12):e10849. 10.15252/emmm.201910849 31633874 PMC6895611

[5] Carafa V Rotili D Forgione M Cuomo F Serretiello E Hailu GS Sirtuin functions and modulation: from chemistry to the clinic. Clin Epigenet (2016) 8:61. 10.1186/s13148-016-0224-3 PMC4879741 27226812

[6] Singh P Hanson PS Morris CM SIRT1 ameliorates oxidative stress induced neural cell death and is down-regulated in Parkinson’s disease. BMC Neurosci (2017) 18(1):46. 10.1186/s12868-017-0364-1 28578695 PMC5455114

[7] Lucena-Cacace A Otero-Albiol D Jimenez-Garcia MP Munoz-Galvan S Carnero A NAMPT Is a Potent Oncogene in Colon Cancer Progression that Modulates Cancer Stem Cell Properties and Resistance to Therapy through Sirt1 and PARP. Clin Cancer Res (2018) 24 p(5):1202–15. 10.1158/1078-0432.CCR-17-2575 29203587

[8] Canto C Menzies KJ Auwerx J NAD(+) Metabolism and the Control of Energy Homeostasis: A Balancing Act between Mitochondria and the Nucleus. Cell Metab (2015) 22(1):31–53. 10.1016/j.cmet.2015.05.023 26118927 PMC4487780

[9] Li XQ Lei J Mao LH Wang QL Xu F Ran T NAMPTand NAPRT, Key Enzymes in NAD Salvage Synthesis Pathway, Are of Negative Prognostic Value in Colorectal Cancer. Front Oncol (2019) 9:736. 10.3389/fonc.2019.00736 31448236 PMC6691178

[10] Zhai RG Zhang F Hiesinger PR Cao Y Haueter CM Bellen HJ NAD synthase NMNAT acts as a chaperone to protect against neurodegeneration. Nature (2008) 452(7189):887–91. 10.1038/nature06721 PMC3150538 18344983

[11] Brazill JM Li C Zhu Y Zhai RG NMNAT: It’s an NAD(+) synthase... It’s a chaperone... It’s a neuroprotector. Curr Opin Genet Dev (2017) 44:156–62. 10.1016/j.gde.2017.03.014 PMC5515290 28445802

[12] Cui C Qi J Deng Q Chen R Zhai D Yu J Nicotinamide Mononucleotide Adenylyl Transferase 2: A Promising Diagnostic and Therapeutic Target for Colorectal Cancer. BioMed Res Int (2016) 2016:1804137. 10.1155/2016/1804137 27218101 PMC4863092

[13] Qi J Cui C Deng Q Wang L Chen R Zhai D Downregulated SIRT6 and upregulated NMNAT2 are associated with the presence, depth and stage of colorectal cancer. Oncol Lett (2018) 16(5):5829–37. 10.3892/ol.2018.9400 PMC6176414 30333863

[14] Sun J Lu F He H Shen J Messina J Mathew R STIM1- and Orai1-mediated Ca(2+) oscillation orchestrates invadopodium formation and melanoma invasion. J Cell Biol (2014) 207(4):535–48. 10.1083/jcb.201407082 PMC4242838 25404747

[15] Jha P Wang X Auwerx J Analysis of Mitochondrial Respiratory Chain Supercomplexes Using Blue Native Polyacrylamide Gel Electrophoresis (BN-PAGE). Curr Protoc Mouse Biol (2016) 6(1):1–14. 10.1002/9780470942390.mo150182 26928661 PMC4823378

[16] Wang J Shen J Zhao K Hu J Dong J Sun J STIM1 overexpression in hypoxia microenvironment contributes to pancreatic carcinoma progression. Cancer Biol Med (2019) 16(1):100–8. 10.20892/j.issn.2095-3941.2018.0304 PMC6528447 31119050

[17] Jing R Corbett JL Cai J Beeson GC Beeson CC Chan SS A Screen Using iPSC-Derived Hepatocytes Reveals NAD(+) as a Potential Treatment for mtDNA Depletion Syndrome. Cell Rep (2018) 25(6):1469–84.e5. 10.1016/j.celrep.2018.10.036 30404003 PMC6289059

[18] Li D Bi FF Chen NN Cao JM Sun WP Zhou YM A novel crosstalk between BRCA1 and poly (ADP-ribose) polymerase 1 in breast cancer. Cell Cycle (2014) 13(21):3442–9. 10.4161/15384101.2014.956507 PMC4613991 25485588

[19] Fang EF Scheibye-Knudsen M Brace LE Kassahun H SenGupta T Nilsen H Defective mitophagy in XPA via PARP-1 hyperactivation and NAD(+)/SIRT1 reduction. Cell (2014) 157(4):882–96. 10.1016/j.cell.2014.03.026 PMC4625837 24813611

[20] Liu TF McCall CE Deacetylation by SIRT1 Reprograms Inflammation and Cancer. Genes Cancer (2013) 4(3-4):135–47. 10.1177/1947601913476948 PMC3764465 24020005

[21] Santolla MF Avino S Pellegrino M De Francesco EM De Marco P Lappano R SIRT1 is involved in oncogenic signaling mediated by GPER in breast cancer. Cell Death Dis (2015) 6:e1834. 10.1038/cddis.2015.201 26225773 PMC4650744

[22] Chowdhry S Zanca C Rajkumar U Koga T Diao Y Raviram R NAD metabolic dependency in cancer is shaped by gene amplification and enhancer remodelling. Nature (2019) 569(7757):570–5. 10.1038/s41586-019-1150-2 PMC7138021 31019297

[23] Ye C Qi L Li X Wang J Yu J Zhou B Targeting the NAD(+) salvage pathway suppresses APC mutation-driven colorectal cancer growth and Wnt/beta-catenin signaling via increasing Axin level. Cell Commun Signal (2020) 18(1):16. 10.1186/s12964-020-0513-5 32005247 PMC6995173

[24] Zhang H Zhang N Liu Y Su P Liang Y Li Y Epigenetic Regulation of NAMPT by NAMPT-AS Drives Metastatic Progression in Triple-Negative Breast Cancer. Cancer Res (2019) 79(13):3347–59. 10.1158/0008-5472.CAN-18-3418 30940661

